# Changes in serum levels of autotaxin with direct-acting antiviral therapy in patients with chronic hepatitis C

**DOI:** 10.1371/journal.pone.0195632

**Published:** 2018-04-04

**Authors:** Tomoo Yamazaki, Satoru Joshita, Takeji Umemura, Yoko Usami, Ayumi Sugiura, Naoyuki Fujimori, Takefumi Kimura, Akihiro Matsumoto, Koji Igarashi, Masao Ota, Eiji Tanaka

**Affiliations:** 1 Department of Medicine, Division of Hepatology and Gastroenterology, Shinshu University School of Medicine, Matsumoto, Japan; 2 Research Center for Next Generation Medicine, Shinshu University, Matsumoto, Japan; 3 Department of Laboratory Medicine, Shinshu University Hospital, Matsumoto, Japan; 4 Bioscience Division, TOSOH Corporation, Ayase, Japan; Nihon University School of Medicine, JAPAN

## Abstract

Sustained virological response (SVR) rates have increased remarkably since the introduction of direct-acting antiviral agents (DAAs) for chronic hepatitis C. Autotaxin (ATX) is a secreted enzyme converting lysophosphatidylcholine to lysophosphatidic acid and a newly established biomarker for liver fibrosis. Interferon-free DAA regimens for chronic hepatitis C could improve liver stiffness in SVR patients according to several non-invasive evaluation methods, but the clinical response and significance of ATX in this context have not yet been defined. We therefore investigated sequential serum ATX levels at baseline, 4 weeks after the start of treatment, and 24 weeks after treatment in 159 hepatitis C virus (HCV)-infected patients who received DAA therapy. Other non-invasive fibrosis markers (aspartate aminotransferase-to-platelet ratio and FIB-4 index) were examined as well. Baseline median ATX levels were comparable between the 144 patients who achieved a SVR and the 15 who did not (1.54 vs. 1.62 mg/L), but median ATX levels became significantly decreased during and after DAA therapy in the SVR group only (from 1.54 to 1.40 and 1.31 mg/L, respectively; *P* < 0.001). ATX was significantly decreased between baseline and 4 weeks of treatment in overall, male, and female SVR patients (all *P* < 0.001). In subjects with low necroinflammatory activity in the liver (i.e., alanine aminotransferase < 30 U/L), ATX levels were significantly reduced from baseline to 4 weeks of treatment and remained low (*P* < 0.001) in patients with a SVR. Thus, interferon-free DAA therapy was associated with a significant decrease in serum ATX levels in patients achieving a SVR, suggesting early regression of liver fibrosis in addition to inflammation treatment.

## Introduction

Persistent hepatitis C virus (HCV) infection develops into chronic hepatitis and leads to cirrhosis and hepatocellular carcinoma (HCC) [1, 2]. Successful HCV eradication, defined as a sustained virological response (SVR), is therefore considered important in decreasing the incidence of HCC [3]. Several new interferon (IFN)-free direct-acting antiviral agent (DAA) regimens have been approved for chronic hepatitis C in Japan [4–6] and have achieved SVR rates of 90–100%, shorter treatment periods, and lower rates of adverse effects. Moreover, accumulating evidence has indicated that IFN-free DAA therapy improves liver fibrosis according to several non-invasive evaluation methods [7–12].

Autotaxin (ATX) plays an important role in converting lysophosphatidylcholine to the bioactive phospholipid lysophosphatidic acid (LPA) [13] involved in physiological roles [14, 15]. As ATX is rapidly taken up by liver sinusoidal endothelial cells [16], reduced clearance of ATX by the damaged or fibrotic liver may explain the elevated serum ATX levels found in patients with liver fibrosis [17]. Serum ATX levels in women are also significantly higher than in men [18, 19] for still unclear reasons, so it is recommended that ATX be assessed by gender. Serum ATX is correlated with liver fibrosis and represents a new non-invasive indicator of hepatic status [18–23]. Although changes in ATX have been studied in small cohorts of HCV-infected patients receiving IFN-free DAA therapy [24], its ability to reflect fibrosis improvement remains unknown. This study therefore assessed the sequential changes in serum ATX levels for evaluating liver fibrosis in patients with chronic hepatitis C before, during, and after IFN-free DAA therapy.

## Materials and methods

### Subjects

Between 2014 and 2016, a total of 159 patients with chronic hepatitis C who received IFN-free DAA therapy (daclatasvir and asunaprevir [n = 61], sofosbuvir/ledipasvir [n = 54], or sofosbuvir and ribavirin [n = 44]) were enrolled in this study. The diagnosis of chronic hepatitis C was based on the presence of serum HCV antibodies and detectable viral RNA, as reported previously [25]. All patients were negative for hepatitis B surface antigen and antibodies to the human immunodeficiency virus. Other causes of chronic liver disease were excluded. Serum levels of HCV RNA were measured with the COBAS TaqMan HCV Test (Roche Diagnostic Systems, Tokyo, Japan). HCV genotypes were determined as described elsewhere [26]. No patient had a history of or developed decompensated cirrhosis or HCC. Cut-off values (mg/L) for ATX levels for each fibrosis stage were determined previously [22] as F1 = 0.8, F2 = 1.1, F3 = 1.3, and F4 = 1.7 for males and F1 = 0.9, F2 = 1.7, F3 = 1.8, and F4 = 2.0 for females. Cirrhosis was observed in 20% (14/70) of males and 40% (36/89) of females based on ATX levels of greater than 1.7 and 2.0 mg/L, respectively. The study was conducted according to the guidelines of the Declaration of Helsinki and was approved by the ethics committee of Shinshu University School of Medicine (approval number: 3244). Written informed consent was obtained from all subjects.

### IFN-free DAA therapy

A 24-week regimen of daclatasvir and asunaprevir [4] or a 12-week course of ledipasvir and sofosbuvir [6] was administered to patients with HCV genotype 1. In patients with HCV genotype 2, a 12-week regimen of sofosbuvir and ribavirin was given [5]. A SVR was defined as undetectable serum HCV RNA at 24 weeks after completing therapy. The presence of known resistance-associated variants in HCV-NS5A at baseline was tested in genotype 1b patients, who were all found to carry the wild-type NS5A region (i.e., absence of leucine at residue 31 and tyrosine at residue 93). Regardless, these subjects were treated with daclatasvir and asunaprevir therapy since SVR rates were reportedly lower in patients harboring such resistance-associated variants [4]. No patient halted IFN-free DAA therapy due to adverse events. For all patients, serum samples were obtained just before treatment (baseline), at 4 weeks of treatment, and at 24 weeks after treatment. All collected samples were immediately stored at -20°C until testing.

### Detection of ATX

We measured ATX levels by a specific two-site enzyme immunoassay using the commercial automated immunoassay analyzer AIA-system (Tosoh Co., Tokyo, Japan) for serum samples obtained before the start of treatment, at 4 weeks after the start of treatment, and at 24 weeks after treatment completion. The AIA-system includes automated 10-microliter specimen dispensation, incubation of the reaction cup, a bound/free washing procedure, 4-methylumbelliferyl phosphate substrate dispensation, fluorometric detection, and a result report. The antigen-antibody reaction time is 10 min and the first result is reported within 20 min. The throughput of the AIA-2000 system is 200 samples/h [27]. The assay was conducted according to the optimized protocols used in previous studies [22, 23].

### Liver fibrosis indices

We examined 2 additional surrogate blood indices of liver fibrosis (FIB-4 index and aspartate aminotransferase [AST]/platelet ratio [APRI]) before treatment, at 4 weeks of treatment, and at 24 weeks after treatment. FIB-4 index and APRI were respectively calculated as: age (years) × AST (U/L)/platelet count (10^9^/L) × alanine aminotransferase (ALT) (U/L)^1/2^ [28] and (AST/upper limit of normal AST [U/L]) × (100/platelet count [10^9^/L]) [29].

### Statistical analysis

Continuous variables are expressed as the median and interquartile range. The Friedman and Wilcoxon signed-rank tests were used to analyze differences among continuous variables at indicated time points. The significance of associations was evaluated by χ^2^ analysis or Fisher’s exact test. A *P* value of less than 0.05 was considered statistically significant. Association strength was estimated by calculating the odds ratio and 95% confidence interval.

## Results

The cohort’s characteristics are summarized in Table 1. Of the 159 subjects, 70 (44%) were male. Median (interquartile range) age was 69 (62–76) years and median ATX level was 1.54 (1.13–2.02) mg/L. Since median ATX levels were higher in women than in men (1.81 vs. 1.33 mg/L; *P* < 0.001), we analyzed our baseline clinical characteristics according to gender. Male patients had significantly higher body mass index, serum creatinine, and hemoglobin values.

**Table 1:** Baseline characteristics of 159 patients with chronic hepatitis C.

	All (n = 159)	Male (n = 70)	Female (n = 89)	*P* value
Age (years)	69 (62–76)	68 (61–73)	72 (65–76)	0.072
Body mass index (kg/m^2^)	21.5 (20.1–23.4)	22.3 (20.7–24.2)	21.0 (19.3–22.9)	0.005
Treatment regimen				0.980
Daclatasvir + asunaprevir	61	26	35	
Ledipasvir + sofosbuvir	54	25	29	
Sofosbuvir + ribavirin	44	19	25	
Genotype (1/2)	115/44	51/19	64/25	0.895
HCV RNA (logIU/mL)	6.1 (5.7–6.6)	6.1 (5.8–6.6)	6.1 (5.7–6.4)	0.595
Alanine aminotransferase (U/L)	39 (24–61)	44 (27–70)	34 (23–57)	0.106
Creatinine (mg/dL)	0.73 (0.63–0.85)	0.85 (0.78–0.97)	0.66 (0.58–0.73)	< 0.001
Albumin (g/dL)	4.1 (3.8–4.3)	4.1 (3.9–4.4)	4.0 (3.8–4.3)	0.207
Hemoglobin (g/dL)	13.8 (12.7–15.1)	15.1 (13.3–15.7)	13.2 (12.6–14.0)	< 0.001
Platelets (×10^4^/mm^3^)	15.3 (10.9–19.8)	14.4 (10.6–18.2)	15.4 (11.3–19.7)	0.198
α-fetoprotein (ng/mL)	4.7 (2.8–7.5)	4.5 (2.4–9.3)	4.8 (3.2–7.3)	0.672
FIB-4 index	3.36 (1.88–4.88)	3.30 (2.09–4.80)	3.37 (1.77–5.01)	0.695
APRI	1.06 (0.63–1.80	1.15 (0.77–2.03)	1.03 (0.55–1.72)	0.146
Autotaxin (mg/L)	1.54 (1.13–2.02)	1.33 (0.95–1.61)	1.81 (1.30–2.41)	< 0.001

Values are expressed as the median (interquartile range).

A total of 144 patients achieved a SVR and the remaining 15 suffered a relapse. We detected no significant differences between patients with and without a SVR in relation to clinical background (Table 2). Baseline serum ATX levels did not differ between the SVR and non-SVR groups (1.54 vs. 1.62 mg/L; *P* = 0.967), nor were remarkable differences seen for FIB-4 index or APRI. Among the 70 male and 89 female patients with chronic hepatitis C, 64 (91%) and 80 (90%), respectively, achieved a SVR. Comparisons of baseline clinical characteristics for each gender revealed no significant differences according to SVR status (Tables 3 and 4). Baseline serum ATX levels were also similar in the SVR and non-SVR groups (male: 1.33 vs. 1.35 mg/L; *P* = 0.976, female: 1.79 vs. 1.95 mg/L; *P* = 0.908, respectively). FIB-4 index and APRI did not differ between the SVR and non-SVR groups by gender.

**Table 2:** Baseline characteristics of 144 patients with a SVR and 15 without.

	SVR (n = 144)	Non-SVR (n = 15)	*P* value
Age (years)	69 (62–76)	69 (64–75)	0.915
Male/female	64/80	6/9	0.741
Body mass index (kg/m^2^)	21.5 (20.1–23.4)	20.3 (19.3–22.5)	0.264
Treatment regimen			0.112
Daclatasvir + asunaprevir	53	8	
Ledipasvir + sofosbuvir	50	4	
Sofosbuvir + ribavirin	41	3	
Genotype (1/2)	103/41	12/3	0.485
HCV RNA (logIU/mL)	6.1 (5.8–6.6)	6.0 (5.6–6.5)	0.726
Alanine aminotransferase (U/L)	39 (24–63)	34 (25–60)	0.888
Creatinine (mg/dL)	0.73 (0.63–0.85)	0.68 (0.63–0.82)	0.493
Albumin (g/dL)	4.1 (3.8–4.3)	4.0 (3.8–4.4)	0.821
Hemoglobin (g/dL)	13.8 (12.8–15.1)	13.2 (12.1–14.0)	0.216
Platelets (×10^4^/mm^3^)	15.5 (11.2–19.8)	10.0 (6.3–17.7)	0.120
α-fetoprotein (ng/mL)	4.6 (2.7–7.3)	6.3 (4.3–12.2)	0.131
FIB-4 index	3.22 (1.89–4.79)	3.91 (1.70–6.28)	0.374
APRI	1.05 (0.63–1.78)	1.23 (0.57–2.04)	0.511
Autotaxin (mg/L)	1.54 (1.14–2.02)	1.62 (1.07–2.06)	0.967

Values are expressed as the median (interquartile range).

**Table 3:** Baseline characteristics of 64 male patients with a SVR and 6 without.

	SVR (n = 64)	Non-SVR (n = 6)	*P* value
Age (years)	67 (57–73)	70 (67–82)	0.210
Body mass index (kg/m^2^)	22.3 (20.8–24.5)	21.0 (20.0–22.0)	0.332
Treatment regimen			0.884
Daclatasvir + asunaprevir	23	3	
Ledipasvir + sofosbuvir	23	2	
Sofosbuvir + ribavirin	18	1	
Genotype (1/2)	46/18	5/1	0.546
HCV RNA (logIU/mL)	6.1 (5.8–6.7)	6.0 (5.2–6.5)	0.417
Alanine aminotransferase (U/L)	44 (27–71)	31 (26–48)	0.260
Creatinine (mg/dL)	0.85 (0.79–0.97)	0.64 (0.64–0.99)	0.416
Albumin (g/dL)	4.2 (3.9–4.4)	4.0 (3.9–4.1)	0.554
Hemoglobin (g/dL)	15.2 (13.7–15.7)	12.3 (12.0–12.5)	0.067
Platelets (×10^4^/mm^3^)	14.7 (10.9–19.6)	8.2 (6.3–10.0)	0.093
α-fetoprotein (ng/mL)	4.5 (2.4–9.2)	6.3 (4.7–10.8)	0.444
FIB-4 index	3.06 (2.01–4.66)	4.43 (3.91–4.93)	0.288
APRI	1.10 (0.77–2.06)	1.35 (0.80–1.76)	0.976
Autotaxin (mg/L)	1.33 (0.97–1.60)	1.35 (0.88–1.69)	0.976

Values are expressed as the median (interquartile range).

**Table 4:** Baseline characteristics of 80 female patients with a SVR and 9 without.

	SVR (n = 80)	Non-SVR (n = 9)	*P* value
Age (years)	72 (65–77)	68 (56–75)	0.297
Body mass index (kg/m^2^)	21.0 (19.3–22.9)	20.3 (18.0–23.0)	0.549
Treatment regimen			0.835
Daclatasvir + asunaprevir	30	5	
Ledipasvir + sofosbuvir	27	2	
Sofosbuvir + ribavirin	23	2	
Genotype (1/2)	57/23	7/2	0.680
HCV RNA (logIU/mL)	6.1 (5.8–6.4)	6.3 (5.6–6.8)	0.799
Alanine aminotransferase (U/L)	33 (22–57)	49 (21–81)	0.518
Creatinine (mg/dL)	0.66 (0.58–0.73)	0.71 (0.57–0.78)	0.714
Albumin (g/dL)	4.0 (3.8–4.3)	4.0 (3.8–4.4)	0.875
Hemoglobin (g/dL)	13.2 (12.6–13.9)	13.7 (12.7–14.4)	0.496
Platelets (×10^4^/mm^3^)	15.8 (11.6–19.8)	13.7 (6.5–20.5)	0.395
α-fetoprotein (ng/mL)	4.6 (3.2–7.2)	6.6 (4.8–11.4)	0.149
FIB-4 index	3.34 (1.77–4.95)	3.82 (1.61–8.23)	0.653
APRI	0.97 (0.54–1.72)	1.09 (0.55–3.73)	0.376
Autotaxin (mg/L)	1.79 (1.31–2.38)	1.95 (1.19–2.79)	0.908

Values are expressed as the median (interquartile range).

**Fig 1:**
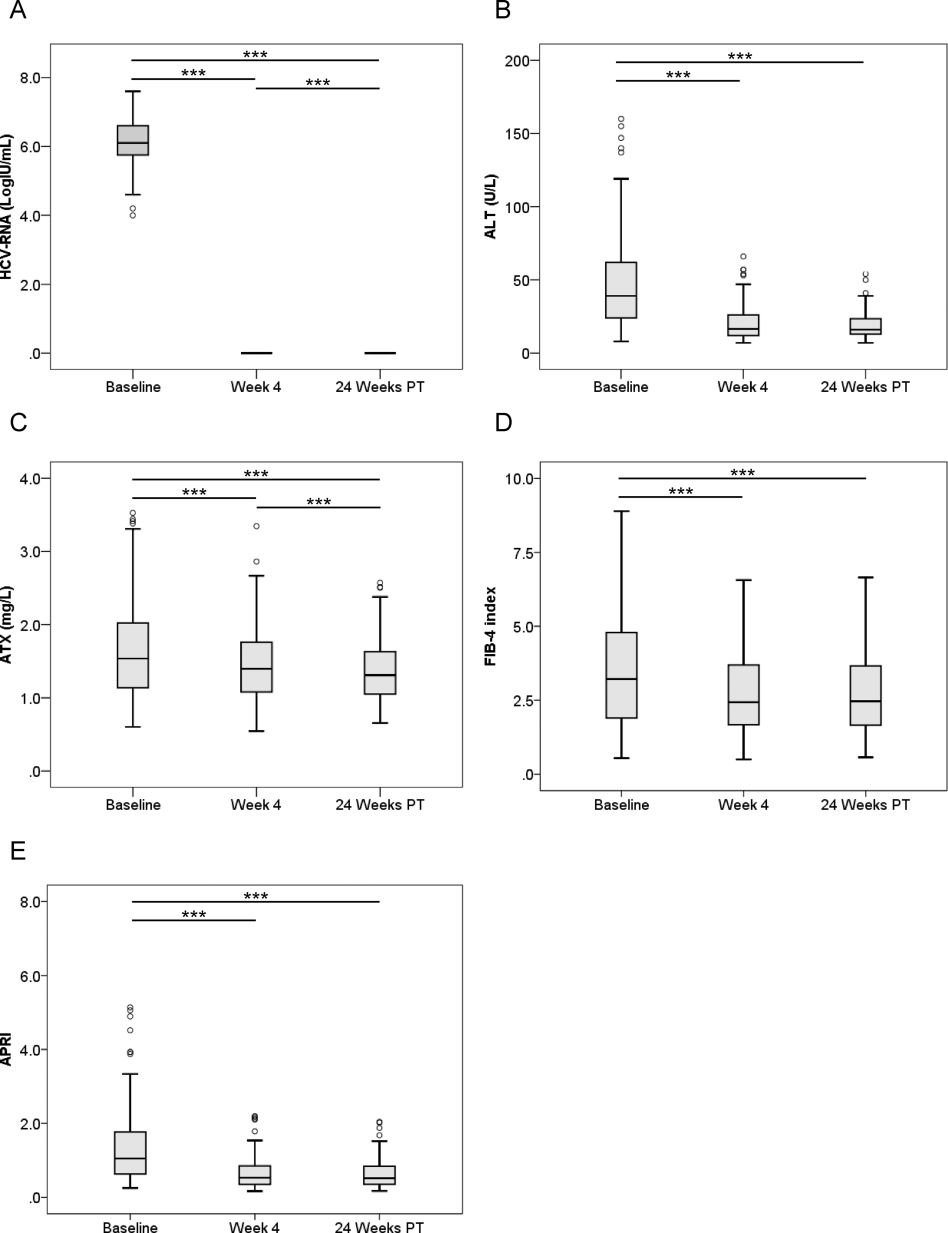
**Changes in serum levels of (A) HCV RNA, (B) ALT, (C) ATX, (D) FIB-4 index, and (E) APRI before, during, and after IFN-DAA therapy in 144 patients with a SVR.** Boxes represent the interquartile range of the data. The line across the boxes indicates the median value. Hash marks depict the nearest value within 1.5 times the interquartile range. Open circles indicate outliers. PT: post-treatment. ***, *P* < 0.001.

**Fig 2:**
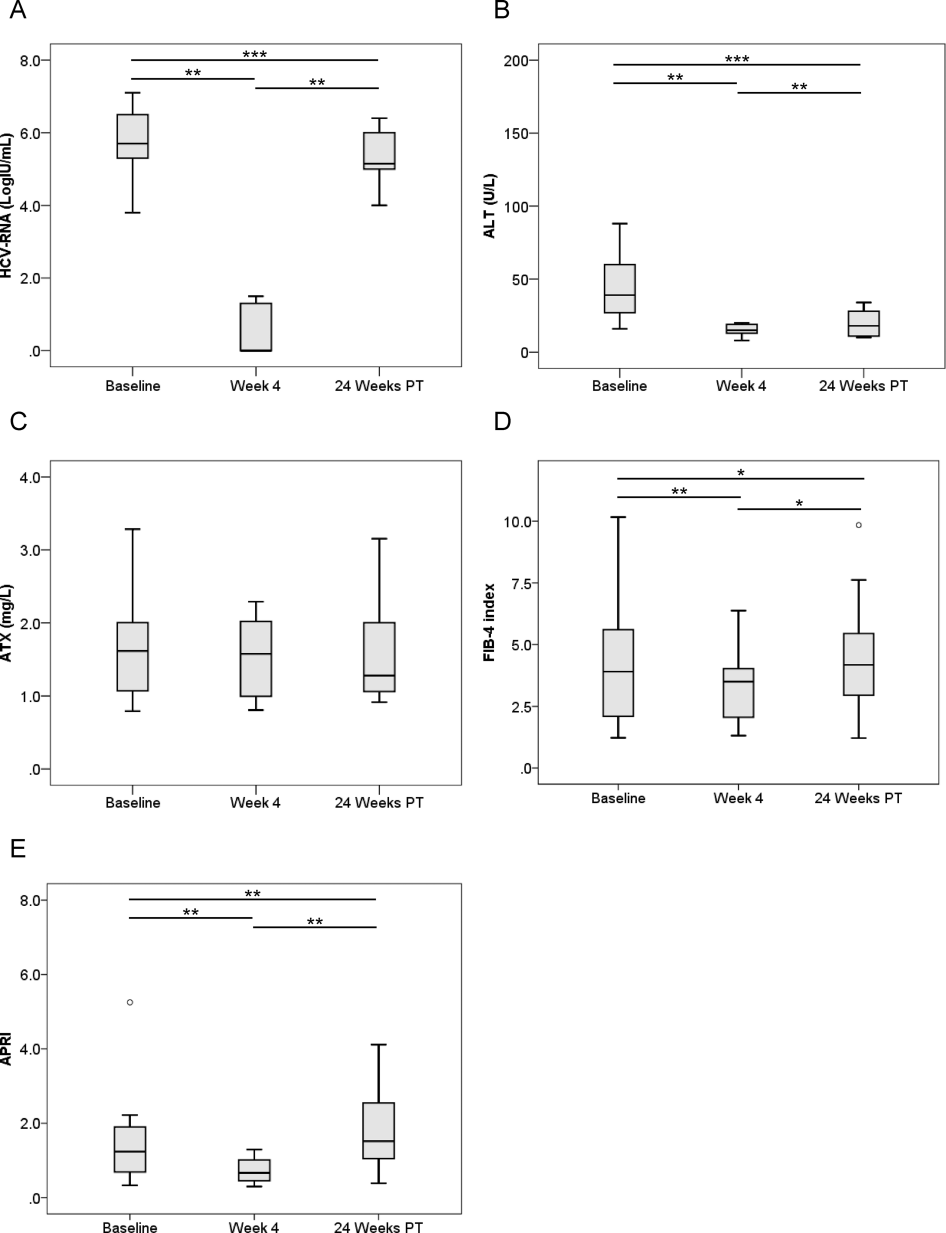
**Changes in serum levels of (A) HCV RNA, (B) ALT, (C) ATX, (D) FIB-4 index, and (E) APRI before, during, and after IFN-DAA therapy in 15 patients without a SVR.** PT: post-treatment. *, *P* < 0.05; **, *P* < 0.01; ***, *P* < 0.001.

We next compared pre-treatment serum ATX levels with those at 4 weeks after the initiation of therapy and 24 weeks after its completion. HCV RNA and ALT levels were significantly decreased overall (*P* < 0.001, Friedman test) (Fig 1A and 1B). Median ATX became significantly decreased in the 144 patients who achieved a SVR (1.54 [1.14–2.02], 1.40 [1.08–1.79], and 1.31 [1.05–1.63] mg/L, respectively; *P* < 0.001) (Fig 1C). There were significant differences between baseline and 4 weeks of treatment (*P* < 0.001) and between 4 weeks of treatment and 24 weeks after treatment (*P* < 0.001). FIB-4 index and APRI were significantly decreased overall (*P* < 0.001, Friedman test) (Fig 1D and 1E). In the 15 patients without a SVR, HCV RNA and ALT levels decreased from baseline to 4 weeks of treatment (*P* < 0.01) and then increased to 24 weeks after treatment (*P* < 0.01) (Fig 2A and 2B). Serum ATX levels decreased gradually, but not significantly (1.62 [1.07–2.06], 1.58 [0.95–2.07], and 1.28 [1.04–2.18], respectively; *P* = 0.058) (Fig 2C). Changes in FIB-4 index and APRI were similar to those for ALT (Fig 2D and 2E).

**Fig 3:**
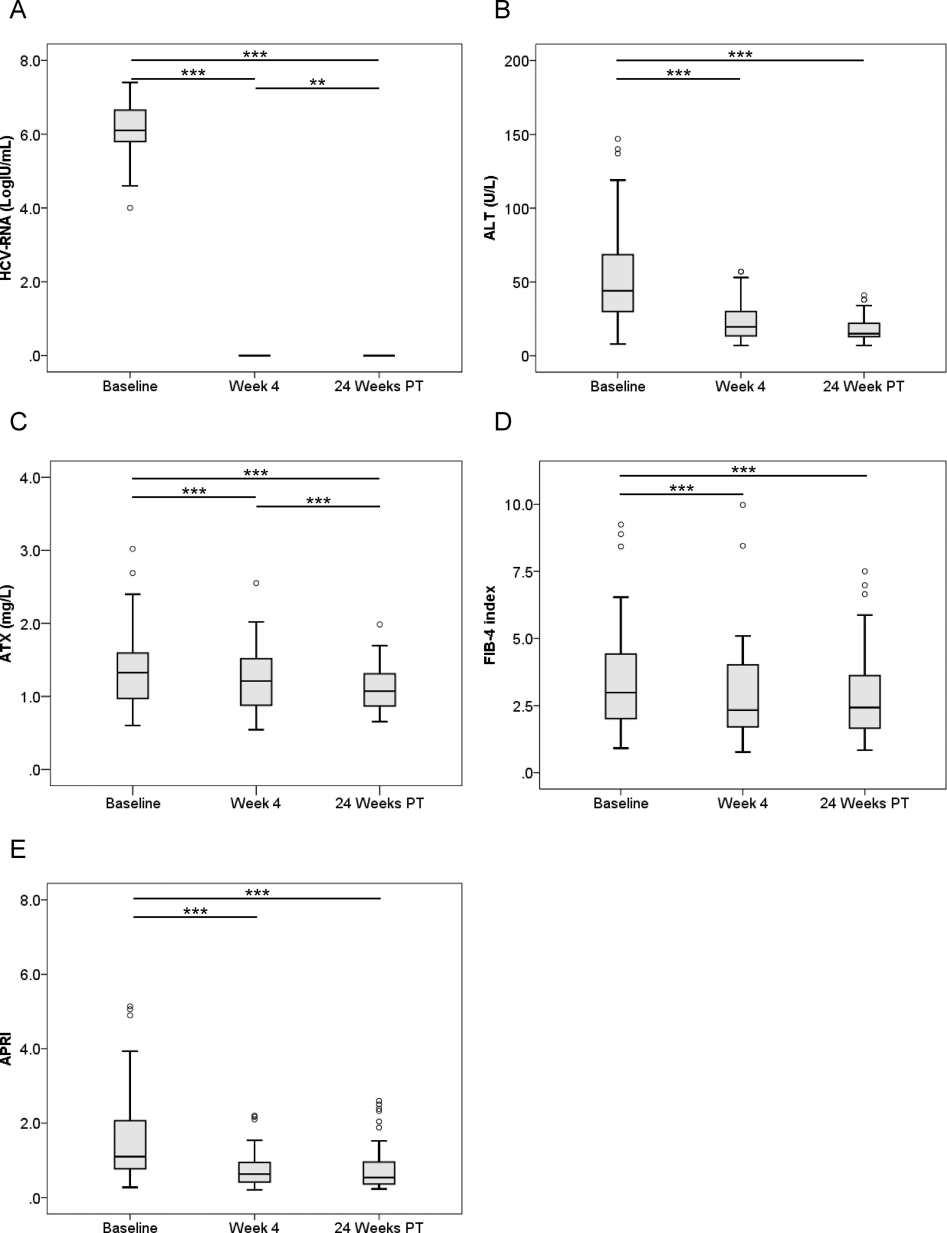
**Changes in serum levels of (A) HCV RNA, (B) ALT, (C) ATX, (D) FIB-4 index, and (E) APRI before, during, and after IFN-DAA therapy in 64 male patients with a SVR.** PT: post-treatment. **, *P* < 0.01; ***, *P* < 0.001.

**Fig 4:**
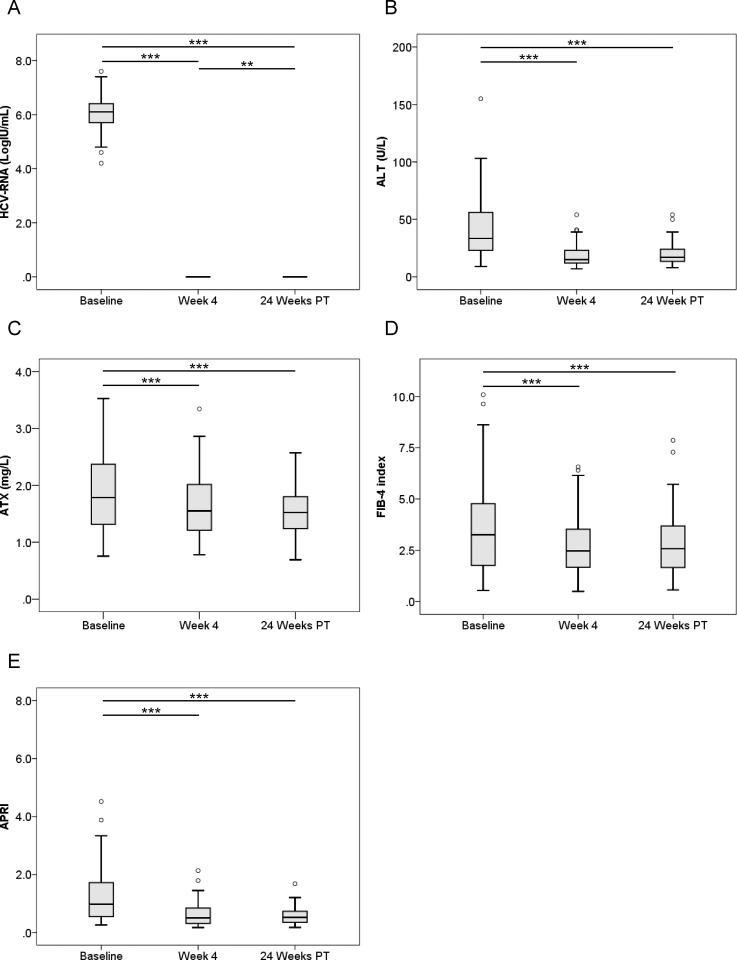
**Changes in serum levels of (A) HCV RNA, (B) ALT, (C) ATX, (D) FIB-4 index, and (E) APRI before, during, and after IFN-DAA therapy in 80 female patients with a SVR.** PT: post-treatment. **, *P* < 0.01; ***, *P* < 0.001.

**Fig 5:**
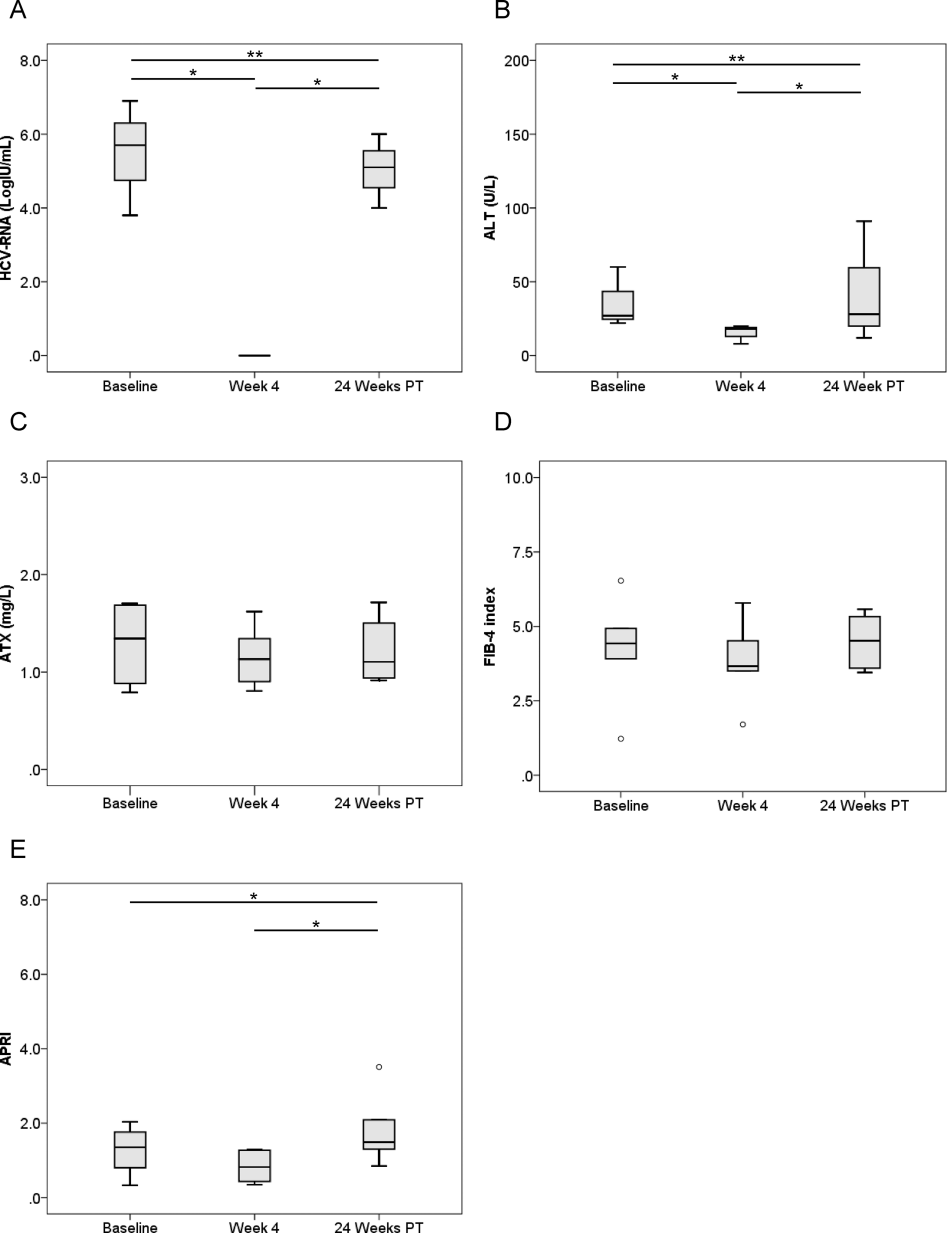
**Changes in serum levels of (A) HCV RNA, (B) ALT, (C) ATX, (D) FIB-4 index, and (E) APRI before, during, and after IFN-DAA therapy in 6 male patients without a SVR.** PT: post-treatment. *, *P* < 0.05; **, *P* < 0.01.

**Fig 6:**
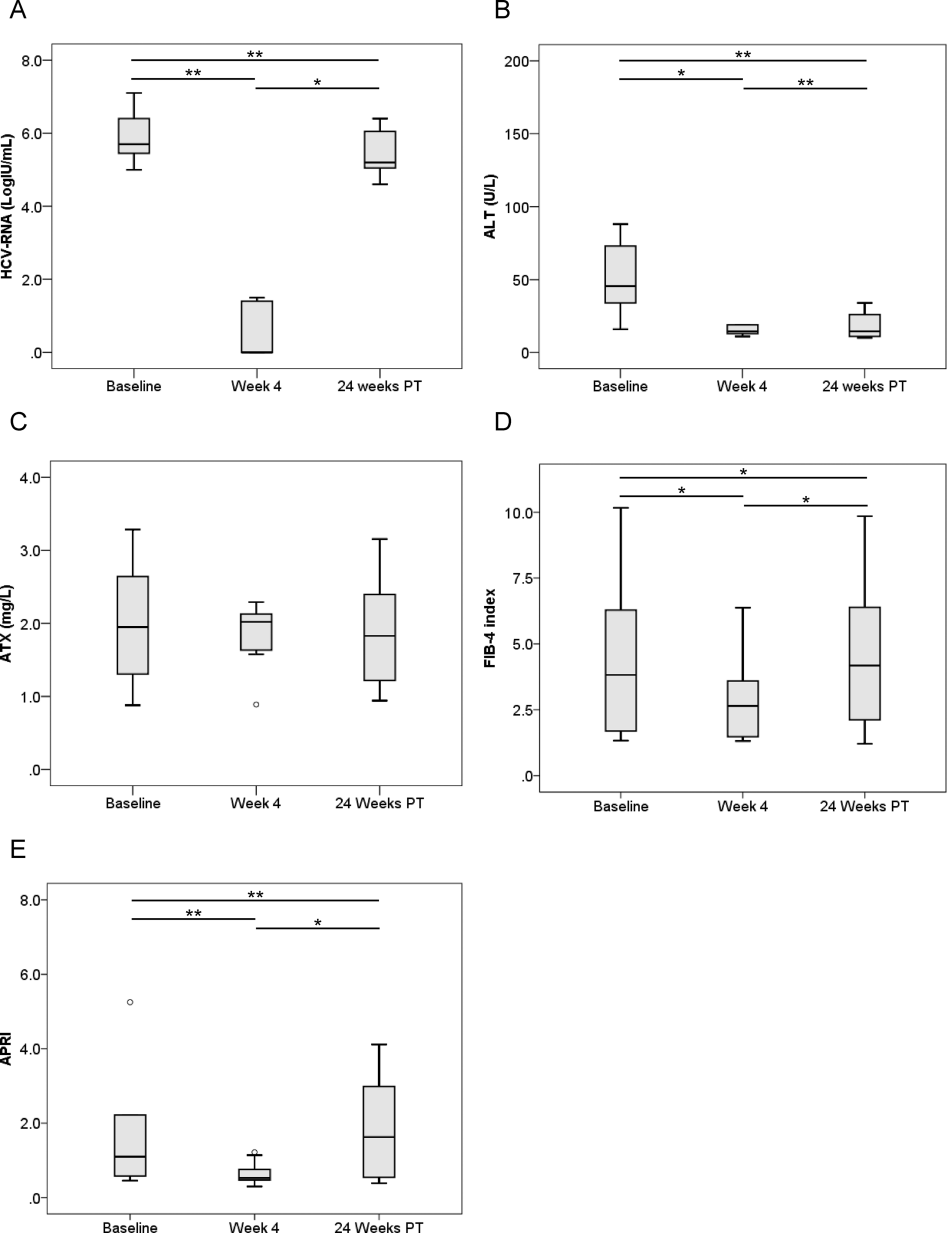
**Changes in serum levels of (A) HCV RNA, (B) ALT, (C) ATX, (D) FIB-4 index, and (E) APRI before, during, and after IFN-DAA therapy in 9 female patients without a SVR.** PT: post-treatment. *, *P* < 0.05; **, *P* < 0.01.

We examined serial HCV RNA, ALT, ATX, FIB-4 index, and APRI changes before, during, and after IFN-free DAA therapy according to gender (Figs 3–6). In male and female patients with a SVR, HCV RNA and ALT levels were significantly decreased overall (Fig 3A and 3B, Fig 4A and 4B). ATX levels became significantly decreased in the 64 male patients (1.33 [0.96–1.60], 1.21 [0.88–1.53], and 1.07 [0.87–1.31], respectively; *P* < 0.001, Friedman test) (Fig 3C) and 80 female patients (1.79 [1.31–2.38], 1.55 [1.20–2.06], and 1.53 [1.24–1.81], respectively; *P* < 0.001) (Fig 4C) achieving a SVR. In particular, ATX significantly decreased from baseline to 4 weeks of treatment in both genders (male: *P* < 0.001, female: *P* < 0.001). FIB-4 index and APRI decreased significantly overall and from baseline to 4 weeks of treatment (Fig 3D and 3E, Fig 4D and 4E). In contrast, HCV RNA and ALT levels decreased from baseline to 4 weeks of treatment and then increased to 24 weeks post-treatment in male and female patients who experienced a relapse (Fig 5A and 5B, Fig 6A and 6B). ATX levels in patients without a SVR did not change significantly during or after therapy in male (1.35 [0.86–1.69], 1.13 [0.88–1.41], and 1.11 [0.93–1.56], respectively; *P* = 0.135) (Fig 5C) or female (1.95 [1.19–2.79], 2.02 [1.58–2.14], and 1.83 [1.15–2.48], respectively; *P* = 0.368) (Fig 6C) subjects. FIB-4 index and APRI decreased significantly from baseline to 4 weeks of treatment and then increased to 24 weeks post-treatment in females (Fig 6D and 6E).

**Fig 7:**
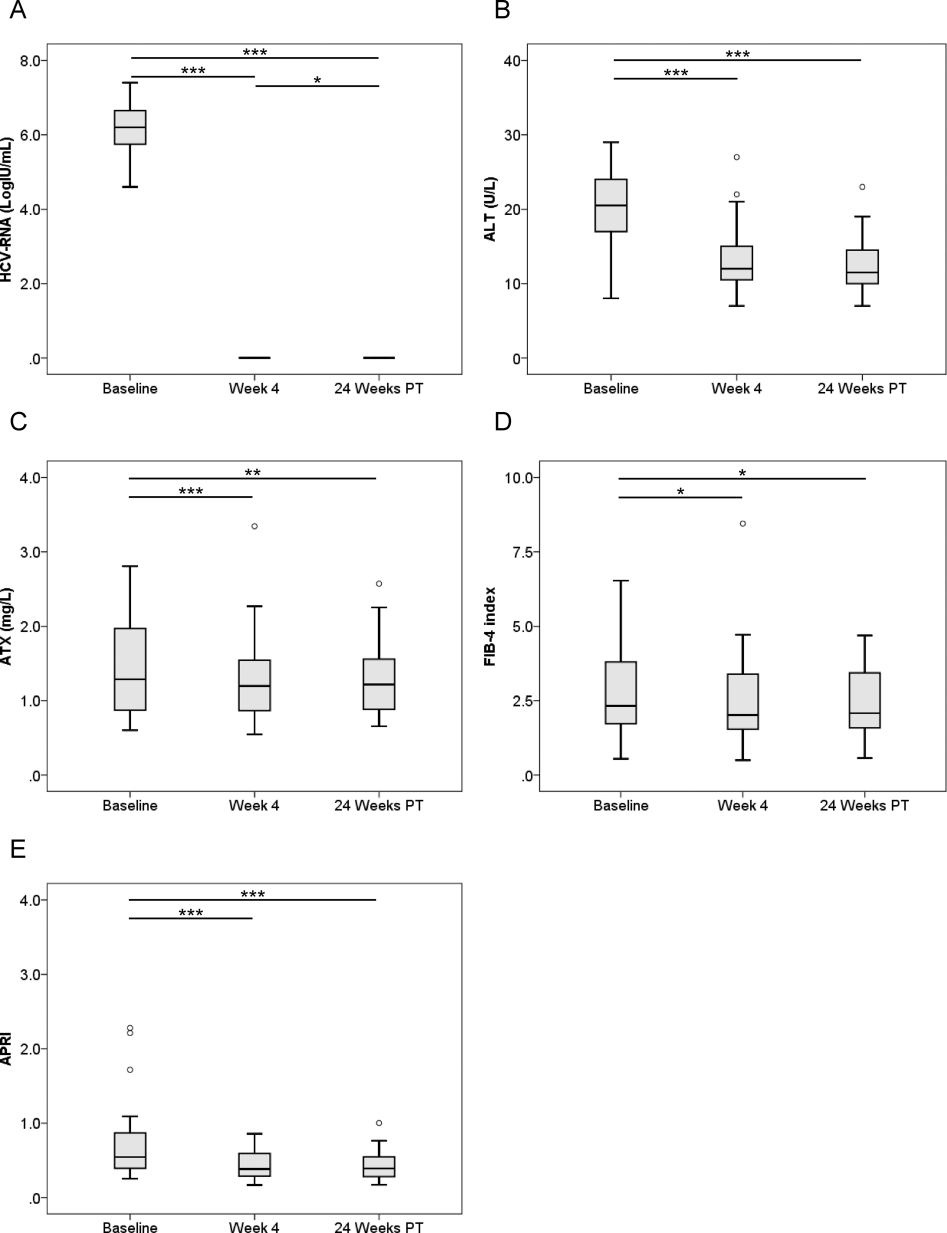
**Changes in serum levels of (A) HCV RNA, (B) ALT, (C) ATX, (D) FIB-4 index, and (E) APRI before, during, and after IFN-DAA therapy in 48 patients with ALT < 30 U/L who achieved a SVR.** PT: post-treatment. *, *P* < 0.05; **, *P* < 0.01; ***, *P* < 0.001.

**Fig 8:**
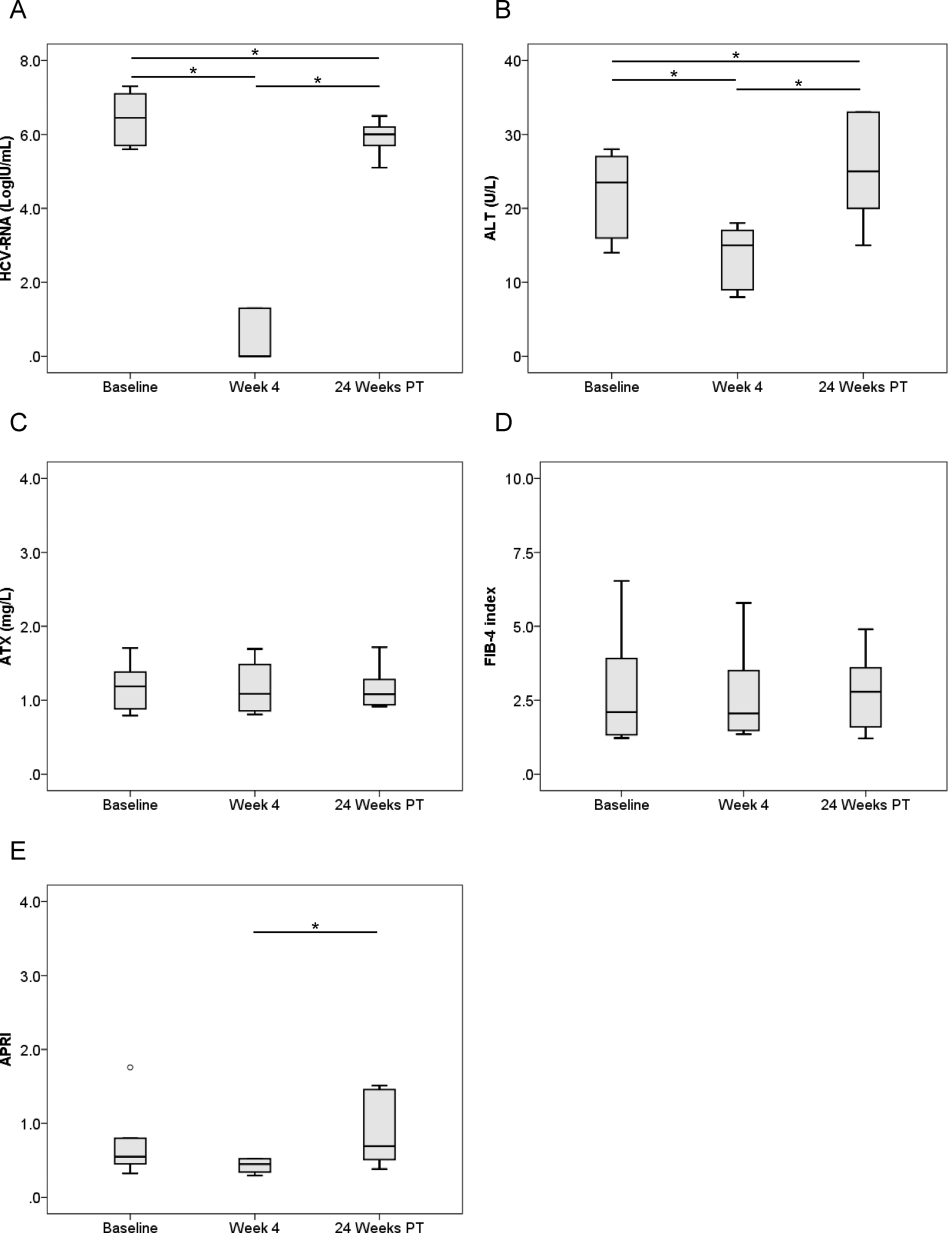
**Changes in serum levels of (A) HCV RNA, (B) ALT, (C) ATX, (D) FIB-4 index, and (E) APRI before, during, and after IFN-DAA therapy in 6 patients with ALT < 30 U/L who did not achieve a SVR.** PT: post-treatment. *, *P* < 0.05.

**Fig 9:**
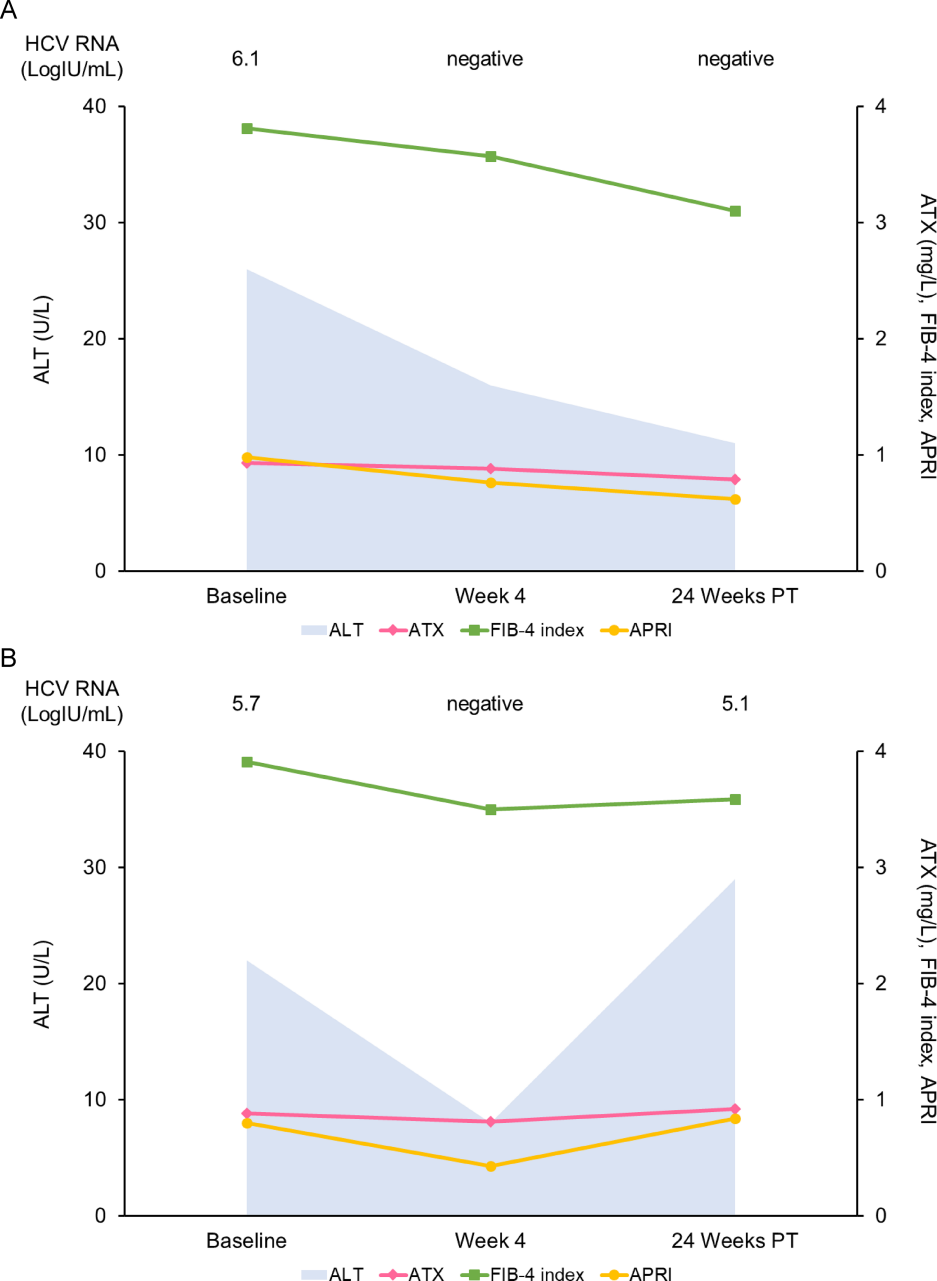
Serial changes in HCV RNA, ALT, ATX, FIB-4 index, and APRI in representative patients with ALT < 30 U/L who were treated with IFN-free DAA therapy. (A) A 71-year-old man was treated with ledipasvir + sofosbuvir for 12 weeks. He achieved a SVR. (B) An 82-year-old man was treated with ledipasvir + sofosbuvir for 12 weeks. He was considered a relapser because HCV RNA was undetectable at 4 weeks of treatment but became positive afterwards. PT: post-treatment.

Lastly, we analyzed the changes in serum ATX of patients with ALT < 30 U/L since ATX has been correlated with necroinflammatory activity in the liver. The clinical characteristics of 54 patients with ALT < 30 U/L are summarized in Table 5. Among 48 SVR patients, serum HCV RNA and ALT levels were decreased significantly overall (Fig 7A and 7B). Serum ATX became significantly decreased over the study period in the 48 patients with a SVR (*P* = 0.002, Friedman test) (Fig 7C),as did overall FIB-4 index and APRI (Fig 7D and 7E). In the 6 patients without a SVR, serum HCV RNA and ALT levels decreased from baseline to week 4 and then increased to 24 weeks post-treatment (Fig 8A and 8B). Serum ATX (*P* = 0.472), FIB-4 index, and APRI were unchanged overall (Fig 8C–8E). The serial changes in HCV RNA, ALT, ATX, FIB-4 index, and APRI were respectively analyzed for representative patients with ALT < 30 U/L and the presence or absence of a SVR (Fig 9A and 9B). There was a general concordance between ALT and viremia levels. Fibrosis markers also correlated with each other in both patients.

**Table 5:** Baseline characteristics of 48 patients with ALT < 30 U/L and a SVR and 6 without.

	SVR (n = 48)	Non-SVR (n = 6)	*P* value
Age (years)	71 (65–78)	69 (58–74)	0.737
Male/female	16/32	3/3	0.420
Body mass index (kg/m^2^)	21.8 (19.8–23.1)	20.3 (19.3–21.3)	0.537
Treatment regimen			0.054
Daclatasvir + asunaprevir	12	5	
Ledipasvir + sofosbuvir	14	1	
Sofosbuvir + ribavirin	22	0	
Genotype (1/2)	26/22	6/0	0.087
HCV RNA (logIU/mL)	6.2 (5.7–6.7)	6.5 (5.7–7.2)	0.510
Alanine aminotransferase (U/L)	21 (17–24)	24 (16–27)	0.426
Creatinine (mg/dL)	0.73 (0.62–0.91)	0.86 (0.63–1.27)	0.406
Albumin (g/dL)	4.1 (3.9–4.3)	4.2 (3.9–4.1)	0.750
Hemoglobin (g/dL)	13.4 (12.4–14.2)	12.5 (11.1–13.9)	0.431
Platelets (×10^4^/mm^3^)	18.2 (13.3–22.3)	17.4 (10.0–25.3)	0.968
α-fetoprotein (ng/mL)	3.5 (2.4–4.7)	3.0 (1.4–12.3)	0.679
FIB-4 index	2.32 (1.70–3.81)	2.10 (1.30–4.56)	0.799
APRI	0.54 (0.39–0.90)	0.55 (0.42–1.04)	0.799
Autotaxin (mg/L)	1.54 (1.14–2.02)	1.62 (1.07–2.06)	0.527

Values are expressed as the median (interquartile range).

## Discussion

The present study examined the serum ATX levels of 159 patients with chronic hepatitis C for associations with treatment outcome of IFN-free DAA therapy. Serum ATX levels were higher in female than in male patients, in agreement with our previous studies [22]. Baseline serum ATX levels were comparable between patients with and without a SVR, but only those achieving a SVR exhibited significantly decreased ATX levels during and after IFN-free DAA therapy in overall, male, female, and low ALT groups.

Although liver biopsy is the gold standard for assessing the degree of liver fibrosis, simple and reliable non-invasive methods are needed to more easily estimate disease and treatment status. After commencing IFN-free DAA therapy, the regression of liver fibrosis in patients with a SVR has been demonstrated by several non-invasive techniques, such as serum hyaluronic acid, type IV collagen, and Wisteria floribunda agglutinin-positive Mac-2-binding protein [7], as well as by transient elastography [8–11], liver and acoustic radiation force impulse [8], and shear wave elastography [12]. We recently evaluated the diagnostic ability of ATX for liver fibrosis in 593 biopsy-confirmed patients with chronic hepatitis C. Serum ATX concentration increased significantly according to liver fibrosis stage both overall and according to gender [22]. Moreover, low serum ATX was significantly associated with longer overall survival in chronic liver disease patients [30]. ATX therefore represents a novel non-invasive biomarker for liver fibrosis and a prognostic indicator of disease activity. However, evidence is scarce on whether and how ATX levels change during and after IFN-free DAA therapy. Kostadinova et al. [24] showed that ATX alterations during HCV infection were reversible within 6 months of initiating IFN-free DAA therapy, but they only included 29 patients with HCV mono-infection and ATX was tested at only 2 points: baseline and week 20–24 of treatment. We therefore increased the number of subjects and analyzed ATX using serial samples. Our data clearly demonstrated that ATX levels became significantly decreased from baseline to 24 weeks post-treatment in patients achieving a SVR. These significant differences were replicated when the patients were stratified according to gender since baseline ATX levels were expectedly higher in women. Although previous reports revealed that ATX was elevated in pregnant women [31] and in patients with follicular lymphoma [32], no such subjects were included in this study. Other factors that possibly increased ATX levels were excluded.

The importance of the ATX-LPA axis has been described for chronic obstructive pulmonary disease, a leading cause of mortality and morbidity [33], in which a selective increase in microRNA-29b in 2 lung compartments in men could potentially account for the observed sex differences in the ATX-LPA axis and its associated pathologies. Moreover, Keune et al. reported that ATX bound to steroids [34], further opening the potential for interactions between sex hormones and the ATX-LPA axis. The above findings highlight the gender differences for ATX and emphasize the importance of sex-stratification in future studies.

ATX levels (Figs 1C, 3C and 4C), FIB-4 index (Figs 1D, 3D and 4D), and APRI (Figs 1E, 3E and 4E) became significantly decreased from baseline to 4 weeks of IFN-free DAA treatment in patients with a SVR both overall and based on gender. Interestingly, serum ALT levels were also significantly decreased during this period in SVR patients (Figs 1B, 3B and 4B). Since our prior study revealed ATX to be correlated with activity grade according to liver biopsy [22], it was probable that the decline in ATX levels reflected an amelioration of necroinflammatory activity. Hence, the continued improvement in ATX levels at 24 weeks post-treatment suggested possible early regression of liver fibrosis in addition to the resolution of inflammation that occurred soon after starting IFN-free DAA therapy. We further examined ATX levels in patients with ALT < 30 U/L having presumably less necroinflammatory activity in the liver. Among the 48 patients who achieved a SVR, we witnessed significantly decreased serum ATX levels over time (*P* = 0.002, Friedman test) and between baseline and 4 weeks of treatment (*P* < 0.001) (Fig 7C). Serum ALT levels became decreased in the early period (*P* < 0.001) and overall (*P* < 0.001) (Fig 7B) and similar decreases in FIB-4 index and APRI were seen (Fig 7D and 7E). In comparisons of serial marker changes in 2 patients with ALT < 30 U/L, there was concordance between ALT level and HCV RNA (Fig 9). Fibrosis markers, such as ATX, FIB-4 index, and APRI levels, were correlated as well (Figs 7–9). Although early improvement of liver fibrosis was likely, we could not reach a definitive conclusion due to a lack of paired histologic findings. Further studies are needed to clarify this observation.

Between 4 weeks of treatment and 24 weeks post-therapy, ATX levels were significantly decreased in all and male patients, but not in female patients. In contrast, ALT, FIB-4 index, and APRI levels did not change in patients with a SVR both overall and based on gender during the same period, suggesting that resolution of fibrosis or inflammation was scarce from 4 weeks of treatment to 24 weeks post-treatment. The reasons for the differences in ATX trends between men and women remain to be clarified.

The ATX-LPA pathway is a suspected regulator of HCC risk in human cirrhosis patients [30, 35]. Based on preclinical findings, treatment with ATX inhibitors and/or LPA receptor antagonists reduced fibrosis in chronic liver disease [30, 35]. Moreover, HCV infection has been shown to increase ATX expression and support viral replication [36]; ATX is therefore a novel player in the pathogenesis of liver fibrosis and HCC and a potential new target for therapy. Investigation of the role of serum ATX in patients with chronic liver disease and HCC has intriguing future prospects.

The main limitations of this study are its retrospective nature and the small number of patients who did not achieve a SVR. Moreover, it will be necessary to compare clinical parameters and biomarkers according to the same regimen and treatment period. Although FIB-4 index and APRI were examined, other liver fibrosis markers, such as hyaluronic acid, type IV collagen, and Wisteria floribunda agglutinin-positive Mac-2-binding protein [37, 38], may also need testing to validate our results in prospective trials of larger cohorts.

In conclusion, our findings implicate a significant decline in serum ATX in patients with chronic hepatitis C who achieve a SVR with IFN-free DAA therapy. Longer follow-up studies are required to determine whether IFN-free DAA therapy improvement of liver fibrosis and reduction of HCC development are reflected in serial ATX measurements.
